# Primary adenocarcinoma of the lacrimal sac with 5-year recurrence-free survival after radiation therapy alone: a case report

**DOI:** 10.1007/s13691-025-00747-0

**Published:** 2025-01-24

**Authors:** Daichi Takizawa, Kayoko Ohnishi, Kentaro Hiratsuka, Ryota Matsuoka, Keiichiro Baba, Masatoshi Nakamura, Takashi Iizumi, Kiyotaka Suzuki, Masashi Mizumoto, Hideyuki Sakurai

**Affiliations:** 1 Department of Radiation Oncology, Hitachi General Hospital, 2-1-1 Jonantyo, Hitachi, Ibaraki 317-0077 Japan; 2 Department of Radiology, School of Medicine, International University of Health and Welfare, 4-3 Kozunomori, Narita, Chiba 286-8686 Japan; 3 Department of Ophthalmology, Hitachi General Hospital, 2-1-1 Jonantyo, Hitachi, Ibaraki 317-0077 Japan; 4 Department of Pathology, Institute of Medicine, University of Tsukuba, 1-1-1 Tennodai, Tsukuba, Ibaraki 305-8577 Japan; 5 Department of Radiation Oncology, Institute of Medicine, University of Tsukuba, 1-1-1 Tennodai, Tsukuba, Ibaraki 305-8577 Japan; 6 Department of Radiation Therapy, Hitachi General Hospital, 2-1-1 Jonantyo, Hitachi, Ibaraki 317-0077 Japan

**Keywords:** Facial neoplasms, Radiation therapy, Lacrimal sac, Aged

## Abstract

Lacrimal sac tumors are rare, with approximately 800 cases reported worldwide; primary adenocarcinoma of the lacrimal sac is particularly rare. Although there is no established treatment strategy, surgical removal is generally performed. However, complete removal often requires extensive resection, including orbital exenteration and lateral rhinoplasty, which is highly invasive and creates significant cosmetic issues. Here, we report our experience with a 72-year-old woman with primary adenocarcinoma of the lacrimal sac with ethmoid bone invasion. She refused surgery and was treated with radiation therapy alone, totaling 70 Gy in 35 fractions. This is the first report of a patient with primary adenocarcinoma of the lacrimal sac who survived for 5 years without recurrence after radiation therapy alone. She experienced late radiation-related complications: the affected eye developed grade-3 retinopathy according to the common terminology criteria for adverse events, version 5.0, and secondary neovascular glaucoma. Cataract and vitreous surgery with retinal photocoagulation were performed. Her cosmetic appearance was maintained after all treatments were completed. Radiation therapy may be an effective treatment for primary adenocarcinoma of the lacrimal sac for patients who either refuse surgery or in whom surgery is not feasible.

## Introduction

Lacrimal sac tumors are rare, with approximately 800 cases reported worldwide [1]. Of the available reports, few are comprehensive, and most are case reports [2, 3]. Between 70 and 80% of lacrimal sac tumors are malignant, with the proportion of malignant tumors being particularly high in elderly patients [1–3]. The most common histologic type of malignant lacrimal sac tumor is squamous cell carcinoma; adenocarcinoma is particularly rare, accounting for 3% to 4% of epithelial tumors [3–6].

Lacrimal sac carcinoma is a progressive, fatal disease that requires early treatment. Treatment usually involves wide surgical resection followed by radiation therapy and/or chemotherapy [1, 7]. However, in order to perform an adequate resection, orbital enucleation or lateral rhinoplasty is often required. These are highly invasive procedures and can cause significant cosmetic issues. According to reports, many patients refuse such invasive surgery [7–9].

We report our experience with an elderly patient with primary lacrimal sac adenocarcinoma. Although surgical resection, including orbital enucleation, was recommended, this patient refused surgery and instead underwent radiation therapy alone, with no evidence of recurrence for 5 years. To the best of our knowledge, there have been no previous reports of long-term recurrence-free survival after radiation therapy alone for primary adenocarcinoma of the lacrimal sac. We performed a literature review to compare our experience with this patient with previous reports of treatment for lacrimal sac adenocarcinoma.

## Case report

**Fig. 1:**
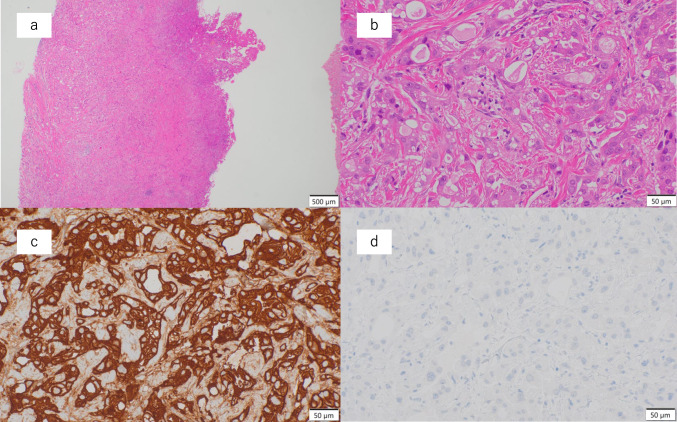
Immunohistochemistry examination. **a**, **b** Hematoxylin–eosin staining shows primary adenocarcinoma of the lacrimal sac. **c** Staining for cytokeratin 7 (CK7) is positive. **d** Staining for thyroid transcription factor 1 (TTF-1) is negative

A 72-year-old woman visited a nearby clinic reporting swelling of the right lower eyelid that had been slowly worsening for about 6 months. Her ophthalmic history included photocoagulation for a right retinal tear and age-related cataracts, which remained untreated. Initially, a stye was suspected and the patient was treated with eye drops. However, her condition did not improve after several months and she was referred to our hospital. Plain magnetic resonance imaging (MRI) revealed a tumor in the right eyelid, and she was referred to a university hospital. A biopsy of the right lower eyelid under local anesthesia revealed adenocarcinoma (Fig. 1a, b). Whole-body examination using positron emission tomography–computed tomography (PET-CT) revealed no abnormal contrast accumulation outside the tumor, ruling out a metastatic tumor.

**Fig. 2:**
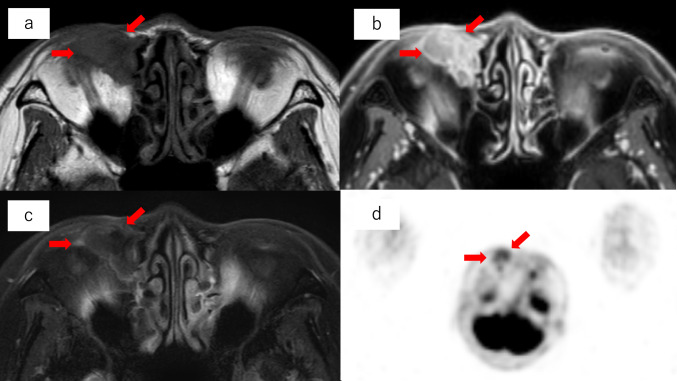
Magnetic resonance imaging (MRI) and positron emission tomography (PET) images before radiation therapy. Red arrows indicate the tumor, a mass with an irregular margin near the right orbit at the medial inferior edge, infiltrating the ethmoid sinus and nasolacrimal duct. **a** T1-weighted MRI demonstrates low signal intensity. **b** Gadolinium (Gd)-enhanced MRI shows high signal intensity. **c** T2-weighted MRI demonstrates low signal intensity. d. PET shows excessive fluorodeoxyglucose (FDG) accumulation with a maximum standardized uptake value (SUVmax) of 2.93, consistent with a tumor

Although the patient had a history of receiving video assisted thoracic surgery for lung adenocarcinoma 13 years prior, the pathologic classification of that tumor was T1aN0M0, stage IA1, according to the 8th edition of the Union for International Cancer Control (UICC) TNM classification. At that time, CT showed a ground-glass opacity measuring about 1 cm, and histopathologic examination revealed a lesion approximately 0.4 × 0.4 cm. The tissue was similar in nature to atypical adenomatous hyperplasia, but due to septal thickening, the patient was diagnosed with adenocarcinoma. Therefore, the possibility of her current tumor being metastatic was considered low. In addition, immunohistochemistry staining of the eyelid biopsy was negative for thyroid transcription factor 1 (TTF-1), a marker for lung adenocarcinoma, and positive for cytokeratin 7 (CK-7), which is typical of adenocarcinoma of the adnexal system. This led us to a diagnosis of primary adenocarcinoma of the lacrimal sac (Fig. 1c, d). Contrast-enhanced MRI revealed a mass measuring 30 mm in diameter that had a low signal on T1- and T2-weighted images but strongly enhanced with gadolinium (Fig. 2a, b, c). PET also showed accumulation of fluorodeoxyglucose (FDG) consistent with a tumor (Fig. 2d). Because the tumor had infiltrated the ethmoid sinus, extensive resection including orbital enucleation and ethmoidectomy was recommended. The patient refused surgery, mainly due to cosmetic concerns, and requested radiation therapy alone.

**Fig. 3:**
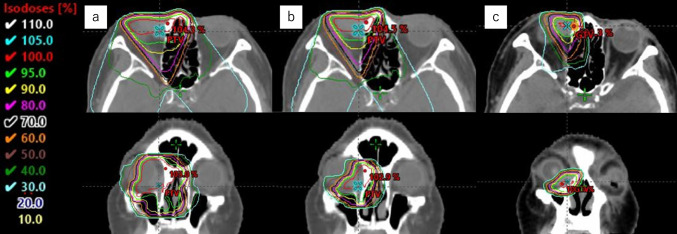
Radiation therapy uses 4 MV x-rays with 3-dimensional conformal radiation therapy (3D-CRT). The initial radiation field encompassed the primary tumor plus a 5-mm margin and the invaded ethmoid sinus. After delivering a dose of 40 Gy in 20 fractions, the radiation field was shrunken to the primary tumor plus a 2-mm margin for a dose of 60 Gy in 30 fractions, followed by a total boost of 70 Gy in 35 fractions to the primary tumor only to reduce the dose to the cornea. **a** 0–40 Gy. **b** 42–60 Gy. **c** 62–70 Gy

**Fig. 4:**
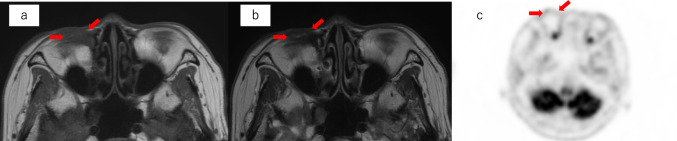
MRI and PET images 4 years after radiation therapy. The red arrows show a smaller tumor. **a** T1-weighted MRI. **b** T2-weighted MRI. **c** PET no longer shows FDG accumulation

She was subsequently treated with 3-dimensional conformal radiation therapy (3D-CRT) with 4 MV x-rays, with a total dose of 70 Gy in 35 fractions (Fig. 3). The maximum dose to the retina was 70 Gy, and the maximum dose to the optic nerve was 43 Gy. At the end of the radiation course, the tumor volume was within the stable-disease range. After radiation was completed, the tumor slowly shrunk over the course of about 1 year. During 5 years of follow up, there has been no evidence of local recurrence, lymph node metastasis, or distant metastasis (Fig. 4).

During the irradiation period, the patient experienced some acute radiation-related adverse events including mild dermatitis, eye discharge, and conjunctivitis, all of which were grade 1 according to the Common Terminology Criteria for Adverse Events (CTCAE), version 5.0. She also had a late radiation-related adverse event: grade 1 eye pain (per CTCAE version 5.0) occurred approximately 1 month after the end of treatment and was still persistent 5 years after finishing radiation therapy. There was no evidence of internal carotid artery stenosis on cervical ultrasound. However, 2 years after completing radiation therapy, right-sided intraocular pressure rose to 37 mm Hg, neovascularization was observed in the angle of the eye, and the patient was diagnosed with radiation-related neovascular glaucoma. Panretinal photocoagulation was performed followed by intravitreal injection of aflibercept, and the intraocular pressure decreased to 21 mm Hg. However, the neovascularization persisted, so she underwent cataract surgery, vitreous surgery, and retinal photocoagulation on the right eye. No complications associated with surgery were observed. After these procedures, normal intraocular pressure was maintained by administering carteolol hydrochloride, latanoprost, brimonidine tartrate, and brinzolamide eye drops.

**Fig. 5:**
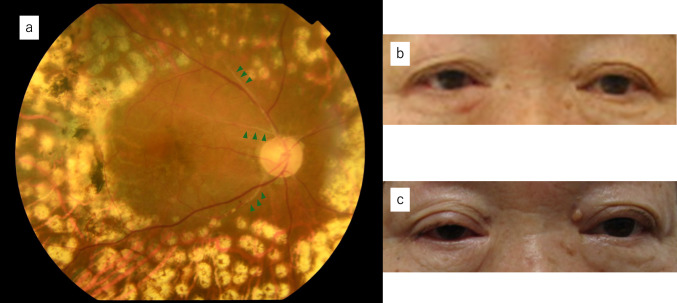
**a** Color fundus photograph taken 5 years after radiation therapy. The green triangles indicate retinal vessel whitening due to retinal artery occlusion. The whitish-gray spots are areas of retinal photocoagulation. **b** External appearance before radiation therapy. **c** There is no noticeable change in external appearance 2 years after radiation therapy

The patient’s corrected vision in the right eye was 20/20 before treatment, but it began to gradually deteriorate about 6 months after completing radiation therapy. Two years later, following the patient’s cataract and vitreous surgery, her corrected visual acuity had decreased to 4/20. Five years after radiotherapy, the corrected visual acuity had decreased to 3/20, and fundus examination revealed retinal artery occlusion, leading to a diagnosis of CTCAE grade 3 radiation retinopathy (Fig. 5a). Although the patient had difficulty judging distances due to a decrease in vision in the right eye, she did not encounter major issues in her daily life.

There were no significant cosmetic changes observed from before irradiation to after the completion of all procedures (Fig. 5b, 5c). The patient was satisfied with her choice of radiation therapy, mainly because of the cosmetic benefits.

## Discussion

Most lacrimal sac tumors are primary tumors and only a few are metastatic [4]. Approximately 75% of primary tumors are epithelial tumors, while the remaining 25% include malignant lymphoma, sarcoma, and malignant melanoma; in either case, a high percentage of tumors are malignant [1, 10, 11]. Most primary epithelial tumors are squamous cell carcinoma, with other types reported including basal cell carcinoma, adenocarcinoma, transitional cell carcinoma, and adenoid cystic carcinoma [1, 3]. Papillary carcinoma and mucoepidermoid carcinoma have a relatively good prognosis, while squamous cell carcinoma, adenocarcinoma, and transitional cell carcinoma reportedly have a poor prognosis [9, 12]. Adenocarcinoma reportedly accounts for only 3% to 4% of primary lacrimal sac epithelial tumors [3–5]; a possible origin of adenocarcinoma of the lacrimal sac is thought to be the seromucinous gland in the lacrimal sac [10, 11, 13].

There is no TNM classification for staging lacrimal sac carcinoma; instead, the classification by Jones et al. is used [14]. Stage 1 is defined as lacrimation, stage 2 as dacryocystitis, stage 3 as painless swelling that does not deform with pressure, and stage 4 as invasion of surrounding tissues. This patient was diagnosed at stage 4. According to the literature, early diagnosis is typically difficult [1, 2]. This is partly because the condition is difficult to differentiate from dacryocystitis based on physical findings in stages 1 and 2. However, MRI effectively differentiates between the 2 entities. As we saw in our patient, lacrimal sac carcinoma is characterized by low signal intensity on T1- and T2-weighted images and high signal intensity with gadolinium contrast. Dacryocystitis appears with high signal intensity on T2-weighted images, making it possible to differentiate between the lesions [15, 16]. If there is any possibility of neoplastic disease, it is advisable to perform MRI proactively.

There is no established method for treating lacrimal sac cancer, but many reports describe aiming for complete surgical resection with the frequent addition of postoperative radiation therapy or chemotherapy. The effectiveness of chemotherapy is unclear from reports to date [1, 3, 4]. The 5-year overall survival rate is about 80% for this type of cancer, with local recurrence reportedly the most frequent type of recurrence [3, 4]. Because of the anatomic structure around the tumor, extensive resection is often required to achieve complete removal. The mortality rate is reportedly significantly lower in patients undergoing extended resection, including lateral nasal resection, than in those undergoing local resection [9], but it is difficult to avoid a significant impact on cosmetic appearance. Radiation therapy is an effective treatment for preserving morphology. Song et al. reported that, in patients with lacrimal sac squamous cell carcinoma who refuse or are unable to undergo surgery, radiation therapy alone or chemoradiotherapy demonstrates overall survival comparable to that of patients who underwent surgery as initial treatment [3, 8].

Because adenocarcinoma of the lacrimal sac is extremely rare, data on outcomes after treatment are limited to case reports. The reports of lacrimal sac adenocarcinoma with described treatment courses are summarized in chronological order in Table 1 [5, 12, 17–25]. In addition to surgery, radiation therapy, and chemotherapy, there are 2 case reports describing the use of hormone therapy for patients with androgen receptor–positive cancer; 1 of these patients achieved long-term progression-free survival with hormone therapy alone [21, 23]. Radiation therapy was administered in 13 of 15 patients. In addition to our patient, Wright et al. reported 4 patients treated with radiation alone, but all 4 experienced recurrence within 2 years [12]. However, all 4 of these patients were treated in the 1970s and 1980s, before the widespread use of 3-dimensional radiation therapy planning devices. Although the total dose and number of fractions was not specified for every patient, most of the described radiation therapy courses used a total dose of 50 Gy or more; the total dose in those cases was lower than in more recent reports [2, 3, 5, 22, 24, 25]. Although the optimal dose is unclear due to the small number of cases of definitive radiation therapy, a higher total dose may improve tumor control. Our patient received a total dose of 70 Gy and achieved 5-year recurrence-free survival. In a report by Okazaki et al., 3-year recurrence-free survival was observed after irradiation with 70 Gy for a locally recurrent tumor 2 years after surgery [25]. The further accumulation of case reports is required to establish the optimal radiation dose.

**Table 1:** Case reports of primary adenocarcinoma of the lacrimal sac

Author (year of publication)	Patient sex, age (y)	Initial treatment	Time of recurrence	Salvage treatment	Radiation dose	Follow-up duration	Outcome
Wright et al. (1992) [12]	M, 47	R	2y	–	No description	3y	DOD
	M, 59	R	2y	–	No description	3y	DOD
	M, 62	R	1y	–	No description	1y	AWD
	M, 45	R	1y	–	No description	1y	AWD
Karim et al. (1997) [17]	M, 48	S	1y	S	–	1y	NED
Baredes et al. (2003) [18]	M, 51	S + R	No recurrence	–	No description	16 m	NED
Brannan et al. (2005) [19]	M, 59	S + R	No recurrence	–	No description	2y	NED
Zhang et al. (2009) [20]	M, 56	S + R	No recurrence	–	No description	2 m	NED
Matsumoto et al. (2014) [5]	F, 79	S + C + R	9 m	–	60 Gy/30 fr	9 m	AWD
Vagia et al. (2015) [21]	M, 65	S + C + R	2y	H	50 Gy	7y	NED
Huggins et al. (2017) [22]	F, 77	S + R	No recurrence	–	60 Gy/10 fr (PBT)	1y	NED
Abramson et al. (2020) [23]	M, 82	H	–	–	–	5y	AWD
Wang et al. (2021) [24]	M, 41	S + R	No recurrence	–	186 Gy (Brachytherapy)	26 m	NED
Okazaki et al. (2021) [25]	F, 65	S	2y	C + R	70 Gy/35 fr	5y	NED
Our patient	F, 72	R	No recurrence	–	70 Gy/35 fr	5y	NED

*M* male, *F* female, *y* years, *R* radiation therapy, *S* surgery, *C* chemotherapy, *H* hormone therapy, *fr* fraction, *PBT* proton beam therapy, *m* months, *DOD* dead of disease, *AWD* alive with disease, *NED* no evidence of disease

Radiation retinopathy is among the causes of secondary neovascular glaucoma, which can lead to blindness. It is believed that radiation damages retinal vascular endothelial cells, causing impaired retinal blood flow and hypoxia, which in turn releases vascular endothelial growth factor (VEGF) and induces neovascularization of the iris and the angle [26]. In general, increasing the total dose of radiation therapy increases the therapeutic effect but also increases the adverse effects on normal tissue. The incidence of radiation retinopathy reportedly increases with increasing radiation dose, rising rapidly when the radiation dose exceeds 50 Gy [26, 27]. Similar to our report, previous reports of radiation therapy for lacrimal sac carcinoma have also noted complications such as decreased vision in the affected eye, eye pain, and neovascular glaucoma [3, 28]. However, in recent reports of radiation therapy for malignant lacrimal sac tumors managed without surgery, a total dose of 60 to 70 Gy has been administered [2, 3, 8, 28]. Reducing the total dose to prevent side effects may increase the risk of recurrence. High-precision radiotherapy, such as intensity-modulated radiation therapy (IMRT), particle therapy, and brachytherapy, is regarded as a method that reduces adverse effects while administering high doses. Previous studies reported the use of highly concentrated brachytherapy and proton-beam therapy as a means of reducing the side effects of radiation; however, only a limited number of facilities provide these treatments [22, 24]. Although few studies have examined the efficacy of IMRT for lacrimal sac carcinoma, in radiation therapy for head and neck cancer, fewer late adverse effects were associated with IMRT than with three-dimensional radiation therapy; therefore, it may also be useful for reducing the adverse effects of radiation therapy for lacrimal sac carcinoma [8, 29].
